# Antioxidant Properties of Novel Lipophilic Fluoroquinolone Compounds Against Oxidative Stress Induced by Acetaminophen and Carbon Tetrachloride in Male Wistar Rats

**DOI:** 10.3390/biom16040567

**Published:** 2026-04-10

**Authors:** Mohammad Alwahsh, Bara’a Shawaqfeh, Rahaf Alejel, Aya Hasan, Dana Yousef, Fadi G. Saqallah, Sameer Al-Kouz, Ameen Alassi, Yasmine Swaiss, Yusuf Al-Hiari, Tariq Al-Qirim

**Affiliations:** 1 Department of Pharmacy, Faculty of Pharmacy, Al-Zaytoonah University of Jordan, Amman 11733, Jordan; b.shawaqfeh@zuj.edu.jo (B.S.); r.alejel@zuj.edu.jo (R.A.); a.hasan@zuj.edu.jo (A.H.); danaazzam19999@gmail.com (D.Y.); f.saqallah@zuj.edu.jo (F.G.S.); s.alkouz@zuj.edu.jo (S.A.-K.); ameen.alassi@zuj.edu.jo (A.A.); yasminswaiss98@gmail.com (Y.S.); tariq.qirim@zuj.edu.jo (T.A.-Q.); 2 Department of Pharmaceutical Sciences, Faculty of Pharmacy, The University of Jordan, Amman 11942, Jordan; yusuf.al-hiari@zuj.edu.jo

**Keywords:** acetaminophen, antioxidant activity, carbon tetrachloride, lipophilic fluoroquinolones, oxidative stress

## Abstract

Oxidative stress is characterised by the production of free radicals in higher amounts than the antioxidant scavenging capacity. This may cause damage to several organs especially the main site of detoxification, the liver. In this study, the antioxidant activity of five novel lipophilic fluoroquinolones (FQs) derivatives was evaluated against oxidative stress induced by acetaminophen (APAP) and carbon tetrachloride (CCl_4_). Sixty-four male Wistar rats were divided into two oxidative-stress models. FQ compounds (25 mg/kg) were administered six hours after CCl_4_ or APAP administration. Serum liver enzymes including aspartate aminotransferase (AST) and alanine aminotransferase (ALT) were measured. Changes in antioxidant parameters were determined in the serum including measurement of total antioxidant status and reduced-glutathione levels as well as catalase, glutathione peroxidase and superoxide dismutase activities. Additionally, molecular docking analyses were performed against catalase, CYP3A4, and Keap-1 to elucidate the potential molecular interactions underlying the observed biological activities. A significant decrease in ALT and AST levels was seen following FQ compound administration in both models. In addition, FQ compounds exhibited excellent antioxidant activity, leading to increased antioxidant enzyme activity, high total antioxidant status, and elevated reduced-glutathione levels. The docking results revealed that compound 4A exhibited the highest binding affinities toward catalase, CYP3A4, and Keap-1. These interactions suggest a possible enhancement of catalase activity, modulation of CYP3A4, and activation of the Keap-1/Nrf2 signalling pathway. Overall, these findings demonstrate the promising therapeutic potential on hepatic injury and oxidative stress of the novel FQ derivatives.

## 1. Introduction

The liver is the main site of metabolism and detoxification, constantly exposed to reactive oxygen species (ROS) [1]. Under physiological conditions, the detoxification process and free-radical generation are in equilibrium. A complex antioxidant defence system is involved in scavenging ROS and counteracting oxidative stress. This system includes enzymatic antioxidants, such as superoxide dismutase (SOD), catalase (CAT), and glutathione peroxidase (GPx), and non-enzymatic antioxidants, such as reduced glutathione (GSH) and ascorbic acid [2]. Together, this system of antioxidants maintains homeostasis between the formation and elimination of free radicals. Oxidative stress occurs when the body is exposed to exogenous or endogenous factors that contribute to the formation of free radicals at rates that surpass scavenging mechanisms [3]. This imbalance in redox homeostasis disrupts signalling pathways, induces inflammation and leads to cellular damage [4]. The accumulation of free radicals can exacerbate the progression of various diseases such as atherosclerosis, diabetes, cancers, neurodegenerative diseases and liver diseases [5,6,7]. Therefore, considering the harmful impact of oxidative stress and its central role in the pathogenesis of many diseases, the discovery and development of new effective antioxidants is a key area of interest.

Acetaminophen (APAP), also commonly known as paracetamol, and carbon tetrachloride (CCl_4_) have been widely used to induce hepatotoxicity and oxidative stress in animal models and their use is well validated in the literature [8,9,10]. APAP, when taken at therapeutic doses, is an effective analgesic and antipyretic drug. However, high doses of APAP have been reported to induce liver damage. This damage is attributed to its excessive formation of APAP’s toxic metabolite N-acetyl-p-benzoquinone imine (NAPQI), which in turn depletes GSH, triggering dysfunction, lipid peroxidation and ROS production [11]. All these consequences of APAP overdose demonstrate the key role that oxidative stress plays in APAP-induced hepatotoxicity. On the other hand, CCl_4_, a well-known hepatotoxin, is metabolised by hepatic cytochrome P 450 enzymes, forming the highly reactive radical trichloromethyl which is also converted into another reactive molecule, the trichloromethyl peroxyl radical. These free radicals covalently bind to DNA, proteins and fatty acids. Targeting polyunsaturated fatty acids initiates lipid peroxidation, leading to cell membrane damage and cellular dysfunction [12]. All these effects induce oxidative stress, inflammation and eventually hepatic injury.

Fluoroquinolones (FQs) are broad-spectrum antibiotics that are effective against aerobic Gram-negative and Gram-positive organisms through the inhibition of bacterial DNA gyrase and topoisomerase IV [13,14]. Any modification will enhance the antibacterial activity and pharmacokinetic properties of the analogue [14]. Certain FQ derivatives have been reported to have antiproliferative activity in human cancer cells [15,16]. Besides their antibacterial and anticancer properties, various studies explored the antioxidant activity of various FQ derivatives [17,18]. One study reported that a number of norfloxacin and ciprofloxacin derivatives containing 1,3,4-oxadiazole, 1,2,4-triazole, and piperazine in their structures exhibited antioxidant activity when assessed using the 2,2- diphenyl-1-picrylhydrazyl (DPPH) assay and cupric-ion-reducing antioxidant capacity [18]. Moreover, the antioxidant activity of a new 2-oxo-1,2-dihydroquinoline-4-carboxylic acid derivative was reported and confirmed by the DPPH, ferric-ion-reducing antioxidant capacity and β-carotene bleaching techniques [19]. This study aims to examine the therapeutic potential of novel lipophilic FQ derivatives in attenuating hepatic injury and their antioxidant activities in dual oxidative-stress models induced by CCl_4_ and APAP.

## 2. Materials and Methods

### 2.1. Chemicals

Dimethyl sulfoxide (DMSO) and ascorbic acid were purchased from Sigma-Aldrich, Burlington, MA, USA. CCl_4_ and APAP were purchased from BDH Chemicals Ltd., Kingston upon Hull, UK.

### 2.2. Synthesis of FQ Derivatives

**Figure 1:**
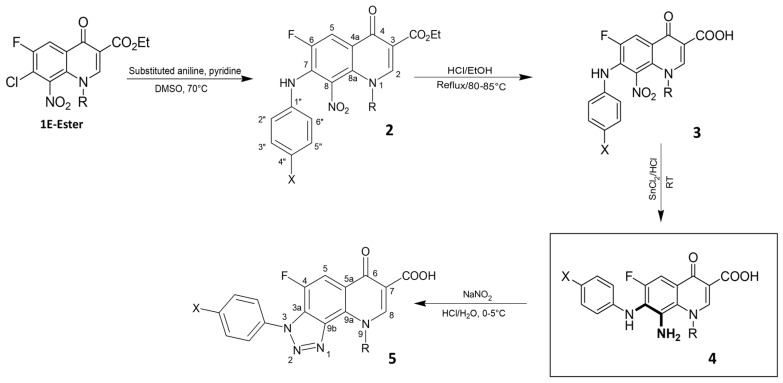
Synthetic pathway of targets **4** (**A**–**E**).

FQ compounds were synthesised and obtained from the Chemistry Laboratory at the University of Jordan [20,21]. Al-Hiari et al. (2025) revealed a formula with excellent antioxidant activity [22]. In this work, novel FQ derivatives had structural features from the previous formula along with new substitutions. Figure 1 illustrates the preparation of the compounds. All FQ compounds were used after checking their purity using nuclear magnetic resonance and mass spectroscopy and using Thin-Layer Chromatography in 94:5:1 chloroform–methanol–formic acid (CHCl_3_-MeOH-FA) mixture. The general structure of each derivative of compound **4** (**A–E**) is detailed in Table 1.

### 2.3. Experimental Design

Sixty-four healthy male Wistar rats weighing 200 ± 10 g were obtained from Al-Zaytoonah University of Jordan. Rats were maintained in 12 h light/dark cycle under constant humidity and room temperature (25 ± 1 °C) with free access to water and food. All animal experiments were ethically approved by the Animal Welfare Committee of the University (#IRB 5/1/2024-2025).

**Figure 2:**
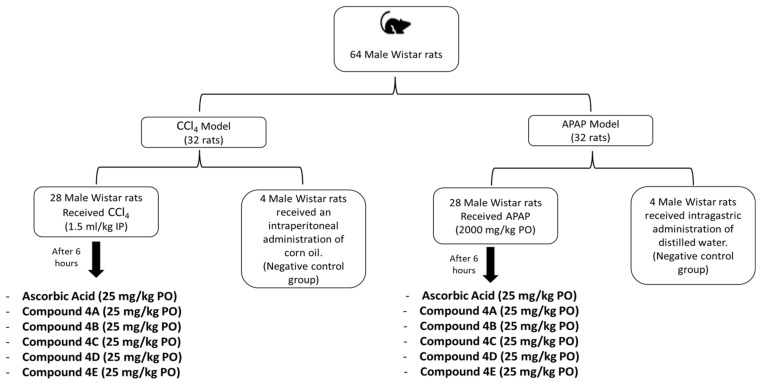
Schematic overview of the experimental design.

The animal experiments included two main oxidative-stress models (CCl_4_ and APAP), allowing for a broader evaluation of the antioxidant effects of the tested compounds. A schematic summary of the experimental design is represented in Figure 2. In the APAP model, overnight-fasted rats were randomly divided into eight groups, each composed of four rats (n = 4). Rats in the negative control group (NCG) received intragastric administration of distilled water. Rats in the APAP model received an intragastric administration of 2000 mg/kg of APAP dissolved in distilled water. Six hours after APAP administration, rats were assigned randomly to treatment groups. FQ compounds groups received an intragastric administration of FQ compounds (**4A**, **4B**, **4C**, **4D**, and **4E**) at a dose of 25 mg/kg dissolved in 3% DMSO. In addition, ascorbic acid was used in this study as the positive control, which was administered by an intragastric gavage at a dose of 25 mg/kg dissolved in 3% DMSO.

In the CCl_4_ model, thirty-two male Wistar rats were divided into eight groups (n = 4). NCG received an intraperitoneal (i.p.) administration of corn oil. Rats in CCl_4_ model were intraperitonially injected with 1.5 mL/kg CCl_4_ dissolved in corn oil (1:1). After six hours of CCl_4_ injection, rats in the treatment groups received an intragastric administration of 25 mg/kg of FQ compounds (**4A**, **4B**, **4C**, **4D**, and **4E**) dissolved in 3% DMSO. Additionally, rats in the ascorbic acid group received an intragastric administration of 25 mg/kg of ascorbic acid dissolved in 3% DMSO. Twenty-four hours following FQ compound administration, rats were anaesthetised and blood was collected from the renal artery, centrifuged at 3000 rpm for 10 min to obtain serum samples and stored at −20 °C for further analysis.

### 2.4. Blood Biochemistry

Analysis of liver enzymes in serum samples was carried out using an enzymatic technique with an automated chemistry analyzer (Model Erba XL-300, Mannheim, Germany).

The alanine aminotransferase (ALT) test was carried out at 37 °C and 340 nm of wavelength using the Erba Manheim^®^/SGPT test Kit. A total of 1 mL of a mixture of reagents R1 (a mixture of tris buffer, L-alanine and lactate dehydrogenase (LDH)) and R2 (a mixture of 3-(Cyclohexylamino)-2-hydroxy-1-propanesulfonic acid (CAPSO), 2-oxoglutarate, and nicotinamide adenine dinucleotide (NADH)) was transferred into a test tube that was then immersed in water at a temperature of 37 °C. Then, 100 µL of the sample was added to the mixture. The absorbance was measured after one, two, and three min. ALT levels were calculated using the following equation:

ALT (IU/L)=(Δabs/min)×1746

The aspartate aminotransferase (AST) test was carried out manually using the Erba Manheim^®^/SGOT test Kit at 37 °C and 340 nm of wavelength. A total of 1 mL of a mixture of reagents R1 (a mixture of tris buffer, L-aspartate, LDH, and malate dehydrogenase) and R2 (a mixture of NADH, CAPSO and 2-oxoglutarate) was transferred into a test tube that was then immersed in water at a temperature of 37 °C. Then, 100 µL of the sample was added to the mixture. Absorbance was measured at one, two, and three min. AST levels were calculated using the following equation:

AST (IU/L)=(Δabs/min)×1746

### 2.5. Determination of Reduced-Glutathione Levels

GSH levels in the serum samples were determined using the reduced glutathione content assay kit (SunLong Biotech, Shanghai, China) following the manufacturer’s instructions. This reaction is based on the ability of glutathione to react with 5,5′-dithiobis-(2-nitrobenzoic acid), producing glutathione disulfide and 2-nitro-5-mercaptobenzoic acid which produces the yellow colour detected at 412 nm. The absorbance was measured at 412 nm and obtained results were calculated based on a standard curve.

### 2.6. Determination of Antioxidant Enzymes Activity

CAT activity was determined using the CAT activity assay kit (SunLong Biotech, China), which is based on the detection of hydrogen peroxide (H_2_O_2_) absorbance at 240 nm. CAT catalyses the breakdown of H_2_O_2_, which lowers the absorbance at 240 nm. Thus, the activity of CAT in the sample can be calculated according to the change in absorbance. The absorbance of the reaction solution was detected at 240 nm using a UV spectrophotometer(Shimadzu, Japan) at the initial time and after 1 min of the reaction. Results are expressed in units/mL.

GPx activity assay was carried out using glutathione peroxidase (GSH-Px/GPX) activity assay kit (SunLong Biotech, China). This method is based on the conversion of GSH to oxidised glutathione by GPx. GSH can react with DTNB to form compounds with distinctive absorption at 412 nm. Higher GPx activity in the sample is indicated by the lowered absorbance at 412 nm. The absorbance of the reaction solution was measured at 412 nm using a microplate reader(Bio Tek, Winooski, VT, USA) and the obtained results are expressed in units/mL.

SOD activity assay was performed using commercially available kit (Genochem World, Spain). This assay is based on the xanthine and xanthine oxidase reaction system, which generates superoxide anion (O2•^−^). In turn, O2•^−^ can reduce blue tetrazole to form blue formazan, which has absorbance at 560 nm. In the presence of SOD, O2•^−^ is converted to oxygen and H_2_O_2_. Therefore, the intensity of the blue colour in this assay is inversely proportional to the SOD activity. The absorbance of the reaction solution was detected using a microplate reader at 560 nm. Results are expressed in units/mL.

### 2.7. Determination of Total Antioxidant Status

The total antioxidant status (TAS) assay was carried out using the total antioxidant capacity (T-AOC) assay kit (Genochem World, Spain) following the manufacturer’s protocol. This assay is performed to detect the total antioxidant levels in the sample. The absorbance of the reaction solution was detected at 593 nm using a microplate reader and the results are expressed in mmol/L.

### 2.8. Molecular Docking

Molecular docking analyses were further made to validate our results. Briefly, the chemical structures of the five FQ analogues were drawn using PerkinElmer ChemDraw 16.0 and their partition coefficient (LogP) values were calculated. Additionally, the chemical structures were geometrically optimised via the Molecular Mechanics 2 (MM2) forcefield using PerkinElmer Chem3D 16.0 [23]. The crystal structures of catalase (CAT; PDB ID: 1DGF) [24], cytochrome P450 3A4 (CYP3A4; PDB ID: 4D6Z) [25], Glutathione Peroxidase-1 (GPx-1; PDB ID: 1GP1) [26], Interleukin-6 (IL-6; PDB ID: 1ALU) [27], Kelch-like ECH-associated protein 1 (Keap-1; PDB ID: 2FLU) [28], and Superoxide Dismutase (SOD; PDB ID: 2C9V) [29] were downloaded from the RCSB Protein Data Bank. All protein structures were cleaned from complexed small molecules and water, and their protonation state was adjusted using BIOVIA Discovery Studio 16 at pH 7.0 [30]. AutoDockTools 1.5.7 was then used for further preparation by adding all hydrogens and assigning Kollman charges to the protein and Gasteiger charges to the compounds [31]. Molecular docking was then performed by using AutoDock Vina 1.2.3 [32,33] for 100 runs with the binding sites’ parameters as illustrated in Table 2. Ascorbic acid was employed as a control molecule against all targets. The results were analysed for the lowest energy of binding (LEB) of each compound towards the targets, while the binding interaction analyses and visualisation were made using BIOVIA Discovery Studio 16 [30].

### 2.9. Statistical Analysis

Statistical analysis of enzyme levels and antioxidant parameters was carried out using GraphPad Prism 8.0.1 software. One-way ANOVA followed by Dunnett’s post-hoc test was used for the comparison between multiple groups. The results were expressed as mean ± SEM and the *p*-value is ^a^ *p* < 0.05 compared with the control group or ^b^ *p* < 0.05 compared with APAP or CCl_4_.

## 3. Results

### 3.1. Effect of FQ Compounds on Liver Injury Markers

**Figure 3:**
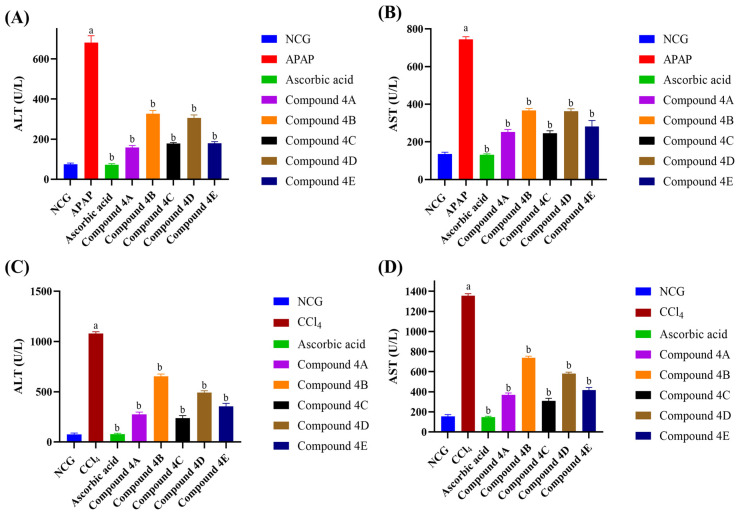
Impact of FQ compounds (4A-4E) on serum liver enzyme levels. (**A**) ALT levels in APAP model, (**B**) AST levels in APAP model, (**C**) ALT levels in CCl_4_ model, and (**D**) AST levels in CCl_4_ model. The bars represent the mean ± SEM of four replicates (n = 4). Symbols indicate significance of differences according to the one-way ANOVA test. ^a^ *p* < 0.05 compared with the control group, ^b^ *p* < 0.05 compared with APAP or CCl_4_. NCG, negative control group; APAP, acetaminophen; CCl_4_, carbon tetrachloride; ALT, alanine aminotransferase; AST, aspartate aminotransferase.

Both APAP and CCl_4_ induced a significant elevation of liver enzyme (ALT and AST) levels compared to the NCG (Figure 3). All FQ compounds and ascorbic acid significantly restored levels of ALT and AST in both models. Compound **4A** showed the highest reduction in ALT levels (76.8%) and compound **4C** showed the highest reduction in AST levels (67%) in comparison to the other compounds in the APAP model as seen in Figure 3A,B. Moreover, among all tested compounds, compound **4C** had the greatest reduction in ALT levels (77.9%) and AST levels (77.2%) compared to the CCl_4_ group as presented in Figure 3C,D.

### 3.2. Effect of FQ Compounds on Reduced Glutathione

**Figure 4:**
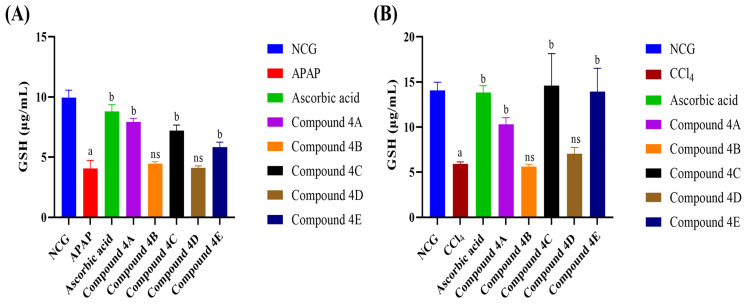
Impact of FQ compounds (4A–4E) on reduced-glutathione levels in the serum of rats in (**A**) APAP model and (**B**) CCl_4_ model. The bars represent the mean ± SEM of four replicates (n = 4). Symbols indicate significance of differences according to the one-way ANOVA test. ^a^ *p* < 0.05 compared with the control group, ^b^ *p* < 0.05 compared with APAP or CCl_4_. ns, non-significant; NCG, negative control group; APAP, acetaminophen; CCl_4_, carbon tetrachloride; GSH, reduced glutathione.

Administration of APAP and CCl_4_ induced a significant reduction in GSH levels (Figure 4). Compounds **4A**, **4C**, and **4E** were able to significantly elevate GSH levels in both models as seen in Figure 4A,B. Among the tested compounds, compound **4A** showed the highest significant increase (95.6%) in GSH levels in the APAP model while compound **4C** has the highest significant increase (147.13%) in GSH levels in the CCl_4_ administration.

### 3.3. Effect of FQ Compounds on Antioxidant Enzymes

**Figure 5:**
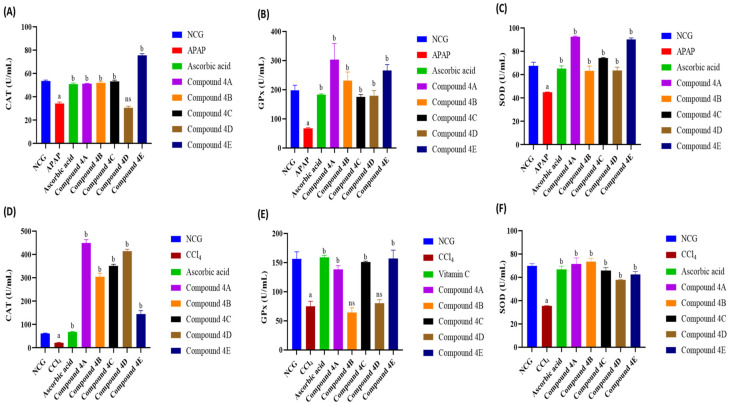
Impact of FQ compounds (**4A**–**4E**) on the enzymatic activities of (**A**) catalase in APAP model, (**B**) glutathione peroxidase in APAP model, (**C**) superoxide dismutase in APAP model, (**D**) catalase in CCl_4_ model, (**E**) glutathione peroxidase in CCl_4_ model, and (**F**) superoxide dismutase in CCl_4_ model. The bars represent the mean ± SEM of four replicates (n = 4). Symbols indicate significance of differences according to the one-way ANOVA test. ^a^ *p* < 0.05 compared with the control group, ^b^ *p* < 0.05 compared with APAP or CCl_4_. ns, non-significant; NCG, negative control group; APAP, acetaminophen; CCl_4_, carbon tetrachloride; CAT, catalase; GPx, glutathione peroxidase; SOD, superoxide dismutase.

Lower antioxidant enzyme activities were observed in the serum of male Wistar rats following APAP and CCl_4_ administration compared to the NCG, as shown in Figure 5. Upon treatment with FQ compounds, this decrease in antioxidant enzyme activities was reversed. In the APAP model, compounds **4A**, **4B**, **4C** and **4E** as well as ascorbic acid showed higher CAT activity compared to the APAP group, with compound **4E** demonstrating the highest significant increase (120%) (Figure 5A). In addition, ascorbic acid and all FQ compounds caused a significant increase in both GPx and SOD in the APAP model. Among the FQ compounds, compound **4A** led to the highest significant elevation in GPx activity (350%) and SOD activity (106.5%) as seen in Figure 5B and Figure 5C, respectively.

Similarly, FQ compounds showed significant effects on antioxidant enzyme activity in the CCl_4_ model. In regard to CAT activity, all tested compound were able to elevate serum CAT activity. Compound **4A** induced the highest significant increase in CAT (1968%) activity as shown in Figure 5D. In addition, compounds **4A**, **4C** and **4E** demonstrated a significant increase in GPx activity (85.25%, 101.6%, and 110%, respectively) comparable to that of the ascorbic acid in CCl_4_ model (Figure 5E). Moreover, while all FQ compounds showed significant increase in SOD activity, compound **4B** exerted the highest elevation (108.3%) (Figure 5F).

### 3.4. Effect of FQ Compounds on Total Antioxidant Status

**Figure 6:**
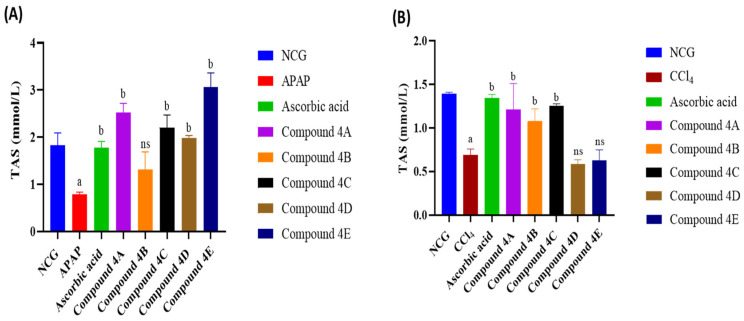
Impact of FQ compounds (4A–4E) on total antioxidant status in the serum of rats in the (**A**) APAP model and (**B**) CCl_4_ model. The bars represent the mean ± SEM of four replicates (n = 4). Symbols indicate significance of differences according to the one-way ANOVA test. ^a^ *p* < 0.05 compared with the control group, ^b^ *p* < 0.05 compared with APAP or CCl_4_. ns, non-significant; NCG, negative control group; APAP, acetaminophen; CCl_4_, carbon tetrachloride; TAS, total antioxidant status.

TAS levels significantly decreased following APAP and CCl_4_ administration (Figure 6). Compared to the APAP group, compounds **4A**, **4C**, **4D**, and **4E** increased TAS by 221.4%, 181.13%, 152.6%, and 291.4%, respectively (Figure 6A). Furthermore, in the CCl_4_ model, compounds **4A**, **4B**, and **4C** showed significant elevation of TAS similar to the ascorbic acid group as seen in Figure 6B.

### 3.5. Molecular Docking

**Figure 7:**
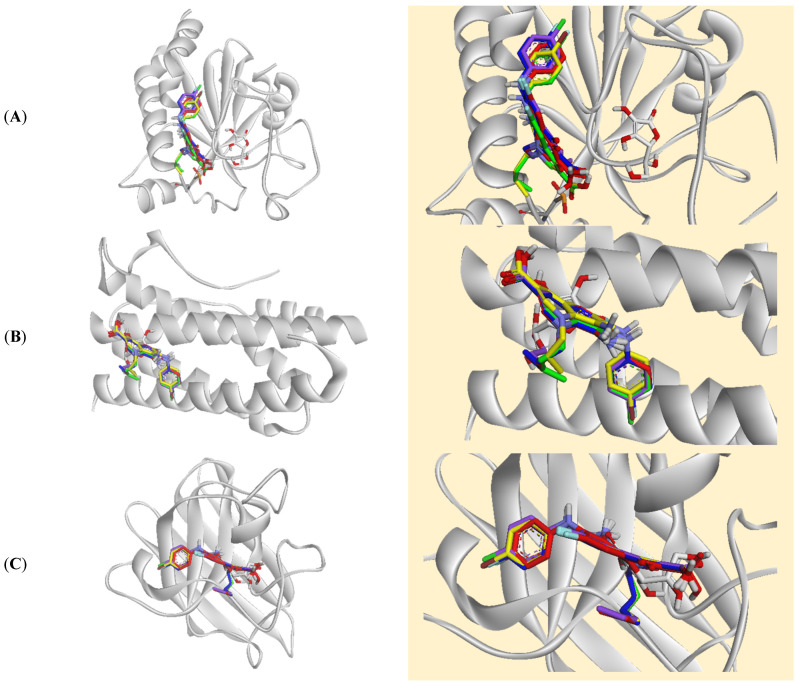
Superimposed stick representation of the five fluoroquinolone analogues (**4A**, red; **4B**, yellow; **4C**, green; **4D**, blue; and **4E**, purple) and ascorbic acid (white) at the binding site of (**A**) glutathione peroxidase-1 (GPx-1), (**B**) interleukin-6 (IL-6), and (**C**) superoxide dismutase (SOD).

Molecular docking simulations were performed to identify the molecular targets through which five FQ analogues exert their antioxidant activities. These simulations were conducted against CAT, cytochrome P450 3A4 (CYP3A4), glutathione peroxidase 1 (GPx-1), interleukin-6 (IL-6), Kelch-like ECH-associated protein 1 (Keap-1), and SOD. Detailed results are summarised in Table S1. Notably, all FQ analogues and ascorbic acid displayed weak binding affinities towards GPx-1, IL-6, and SOD. Rather than dismissing these findings, they warrant mechanistic consideration. The weak docking scores for GPx-1 and SOD do not contradict the significant in vivo elevation of their activities observed in both the APAP and CCl_4_ models. Instead, these findings collectively suggest that the FQ compounds do not exert their effects on GPx and SOD through direct enzyme binding, but rather through an indirect transcriptionally mediated mechanism. Specifically, the strong binding affinities observed at Keap-1 in this study suggest that FQ-mediated disruption of the Keap-1/Nrf2 protein–protein interaction may liberate Nrf2, enabling its nuclear translocation and subsequent antioxidant response element (ARE)-driven transcriptional upregulation of GPx, SOD, CAT, and GSH biosynthetic enzymes. This indirect pathway provides a mechanistically coherent explanation for the discrepancy between the weak docking scores and the robust in vivo antioxidant enzyme activities observed experimentally. The binding poses of the docked compounds towards these proteins are presented in Figure 7, and their binding interactions are illustrated in Figure S1, Figure S2, and Figure S3, respectively.

**Figure 8:**
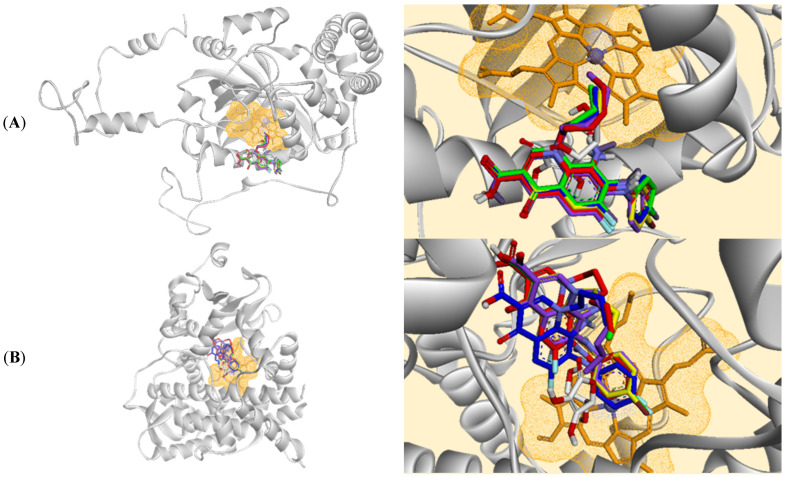
Superimposed stick representation of the five fluoroquinolone analogues (**4A**, red; **4B**, yellow; **4C**, green; **4D**, blue; and **4E**, purple) and ascorbic acid (white) at the binding site of (**A**) human erythrocyte catalase enzyme, and (**B**) cytochrome P450 3A4 (CYP3A4) isozyme with the heme molecule (orange) surrounded by an orange mesh surface.

All FQ analogues demonstrated varying binding affinities and interaction profiles with the CAT enzyme. Figure 8A represents the binding conformations of these compounds at the active site of CAT, while Figure S4 illustrates its binding interactions with the surrounding amino acid residues. Compound **4A** exhibited the highest binding affinity with an LEB of −10.340 kcal/mol, forming two hydrogen bonds with Thr361 and Asp360, one π-alkyl interaction with the heme, and ten hydrophobic interactions. This agrees with the experimental results as compound **4A** demonstrated the highest significant increase in CAT levels following CCl_4_ intervention. Compounds **4B** and **4C** had LEBs of −7.702 and −6.397 kcal/mol, respectively. Both compounds exhibited similar hydrogen bonding and π-alkyl interaction profiles as **4A**, with similar hydrophobic interaction patterns. Compound **4D** showed an LEB of −7.595 kcal/mol, characterised by one hydrogen bond with Thr361, one π-alkyl interaction with the heme, and comparable hydrophobic interactions. Compound **4E**, with an LEB of −7.650 kcal/mol, had two hydrogen bonds with the same residues as **4A**–**4C**, one van der Waals (vdW) interaction with the heme, and similar hydrophobic interactions to the other compounds. Yet, ascorbic acid, the control, exhibited an LEB of −3.830 kcal/mol, featuring two hydrogen bonds with Thr361 and Asp360, one vdW interaction with the heme, and several hydrophobic interactions with Val73, Ala357, Phe161, Pro162, and Phe356.

For CYP3A4, Figure 8B illustrates the binding conformations of the FQ analogues at the binding site of the protein. Herein, compound **4A** demonstrated the highest binding affinity with an LEB of −9.572 kcal/mol, characterised by one hydrogen bond with Arg372 and a π-π stacking interaction with the heme, along with various hydrophobic interactions with 13 residues (Figure S5). Compounds **4B**, **4C**, **4D**, and **4E** had LEB values of −8.782, −8.704, −8.546, and −9.286 kcal/mol, respectively. These compounds lacked hydrogen bond interactions but featured π-cation interactions with the heme and exhibited 12–13 hydrophobic interactions. Still, ascorbic acid showed an LEB of −4.531 kcal/mol, with one hydrogen bond with Phe304, one π-donor hydrogen bond interaction with the heme, and fewer hydrophobic interactions compared to the FQs, involving Ala305, Thr309, Arg212, Ala370, and Phe213.

**Figure 9:**
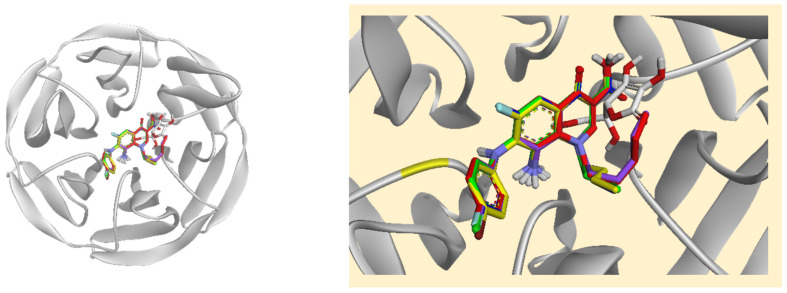
Superimposed stick representation of the five fluoroquinolone analogues (**4A**, red; **4B**, yellow; **4C**, green; **4D**, blue; and **4E**, purple) and ascorbic acid (white) at the binding site of Kelch-like ECH-associated protein 1 (Keap-1).

For Keap-1, Figure 9 illustrates the binding conformations of the FQ analogues at the binding site of the protein. Compound **4A** exhibited an LEB of −7.968 kcal/mol, with four hydrogen bonds with Arg380, Asn414, and Tyr525, alongside numerous hydrophobic interactions with 14 residues (Figure S6). Compound **4B** had a slightly higher binding affinity with an LEB of −8.086 kcal/mol, forming three hydrogen bonds with Arg380, Asn414, and Ser555, and demonstrating a similar range of hydrophobic interactions as Compound **4A**. Compound **4C** had an LEB of −7.481 kcal/mol, characterised by three hydrogen bonds, two of them with Arg380 and one with Ser555, and a comparable hydrophobic interactions profile. Compound **4D** exhibited an LEB of −7.891 kcal/mol, with similar hydrogen bonds and hydrophobic interactions as in **4C**. Compound **4E**, with an LEB of −7.810 kcal/mol, had one hydrogen bond with Arg380 and various hydrophobic interactions with nine residues, such as Ala556, Arg415, Asn414, Arg483, Tyr572, Ser602, Phe577, Tyr334, Asn382, Gly364, Ser363, Gly603, Ser555, Gln530, Tyr525. Nonetheless, ascorbic acid had an LEB of −5.574 kcal/mol with four hydrogen bonds, two of them with Ser363, one with Arg380, and a fourth with Arg415, among various hydrophobic interactions with Tyr334, Asn382, Arg415, Gly364, Asn414, Ala556, Gly603, Ser602, and Ser338.

## 4. Discussion

ROS overproduction and depletion of antioxidant enzymes are the main contributors of the induction of oxidative stress. CCl_4_ and APAP are well-known potent inducers of liver injury and oxidative damage used in the assessment of potential hepatoprotective agents [34]. Various studies have reported that CCl_4_ can cause liver injury and oxidative stress due to its ability to produce free radicals [35,36,37,38]. Following its administration, it is metabolised by cytochrome P450 enzyme 2E1 (CYP450-2E1) isoform to a variety of free radicals such as trichloromethyl radical and peroxy trichloromethyl radical [39]. Consequently, these produced radicals lead to structural changes in the plasma membrane of liver cells (e.g., hepatocytes mitochondria), contributing to oxidative damage [40,41]. A single dose of APAP has been found to induce oxidative stress in a rat model [42]. The mechanisms of its toxicity start with the metabolism of APAP in the liver by cytochrome P450 enzymes, where it is converted to the reactive metabolite NAPQI. When taken in high dose, NAPQI levels exceed detoxification capacity, leading to depleted GSH, leading to oxidative damage [43]. In this study, both CCl_4_ and APAP rat models were employed in the evaluation of the pharmacological activity of novel lipophilic FQ compounds (**4A**, **4B**, **4C**, **4D**, and **4E**) against oxidative stress and their ability to restore antioxidant levels and antioxidant enzyme activities along with liver enzymes.

Liver enzymes levels are used as a marker of hepatocellular damage. Loss of membrane integrity due to liver injury leads to the release of hepatocyte enzymes into the circulation, elevating their levels in the serum [44,45]. Elevation in the levels of ALT and AST enzymes has been reported to be induced by APAP [44,46] and CCl_4_ [47]. In this study, the marked elevation of serum ALT and AST following the administration of APAP and CCl_4_ indicates the induction of liver injury. FQ compounds were able to significantly deplete their levels in both APAP and CCl_4_ models. Among the tested compounds, compounds **4A** and **4C** showed the most marked reduction. These findings demonstrate the potential pharmacological activity of the tested compounds against liver injury.

The body has multiple defence mechanisms that together work within a complex antioxidant defence system to protect it from oxidative stress, preventing the accumulation of free radicals [48,49]. This system consists of both enzymatic and non-enzymatic antioxidants. CAT, GPx, and SOD represent the major antioxidant enzymes that function together to protect cells from ROS [50,51]. SOD is an endogenous antioxidant enzyme that drives the conversion of O2•^−^ into H_2_O_2_, reducing the threat of the superoxide anion [52]. H_2_O_2_ is subsequently converted into water by CAT and GPx, thus completing the detoxification process [51]. In this study, oxidative stress induced by APAP and CCl_4_ was marked by the decreased activities of CAT, GPx, and SOD and lower TAS and GSH levels. Previous studies have reported decreased antioxidant parameters in rats treated with APAP [46,53] and CCl_4_ [35,54,55]. Lipophilic FQ derivatives were able to reverse these changes, restoring the levels of antioxidants and increasing the activities of antioxidant enzymes. These effects can enhance the antioxidant defence mechanisms, reduce the accumulation of free radicals, and ameliorate oxidative damage induced by CCl_4_ and APAP. Taken together, FQ derivatives exhibited ameliorative effects of liver injury, reducing ALT and AST levels comparably to ascorbic acid. In addition, among the five tested derivatives, compounds **4A**, **4C** and **4E** showed the most promising effects in both models, exhibiting superior activity in improving non-enzymatic and enzymatic antioxidants following the induction of oxidative stress compared to the standard antioxidant ascorbic acid. Notably, compound **4A** effectively increased GPx and SOD activities in the APAP model as well as improved CAT activity in the CCl_4_ model. Compound **4C** showed a pronounced effect on GSH levels in the CCl_4_ model, improving the availability of this endogenous antioxidant in the serum. Compound **4E** exhibited excellent efficacy, greatly increasing GSH levels and GPx activity in CCl_4_ model and enhancing the activities of GPx, CAT and SOD in the APAP model. Moreover, compounds **4A**, **4C** and **4E** effectively increased TAS levels in the APAP model, exceeding the effect observed with ascorbic acid, which indicates a strong improvement of the antioxidant defence. These findings illustrate the promising activity of compounds **4A**, **4C** and **4E**, suggesting their therapeutic potential as more effective antioxidant agents. The therapeutic potential against hepatic injury of novel FQ derivatives and their antioxidant activity was confirmed in both models, suggesting that these compounds may exert their effects through different mechanisms of action, due to differences in the mechanisms between CCl_4_ and APAP in the production of free radicals.

Although FQ compounds demonstrated significant in vivo elevation of GPx and SOD activities and GSH levels, the molecular docking simulations revealed weak binding affinities of all tested compounds and ascorbic acid toward GPx-1, SOD, and IL-6. This apparent discrepancy between the in vivo and in silico findings can be mechanistically resolved. Molecular docking evaluates direct protein–ligand interactions under static, computationally isolated conditions, and therefore does not capture indirect or transcriptionally mediated mechanisms of enzyme activation. The observed restoration of GPx and SOD in vivo activities is most likely not attributable to direct enzyme binding, but rather to upstream transcriptional induction mediated through the Keap-1/Nrf2/ARE signalling axis. Nrf2, once liberated from Keap-1-mediated proteasomal degradation, translocates to the nucleus and drives the expression of a broad battery of cytoprotective genes, including those encoding GPx, SOD, CAT, and enzymes involved in GSH biosynthesis such as glutamate–cysteine ligase [56,57,58]. This mechanistic interpretation is consistent with the strong binding affinities of the FQ compounds at Keap-1 observed in this study, and provides a unifying explanation for the collective restoration of all antioxidant parameters measured experimentally.

Through molecular docking, the binding affinities of the FQ compounds towards human erythrocyte catalase suggest that these compounds might enhance catalase activity, which is crucial for cellular protection against oxidative damage. The negative binding energies indicate favourable binding interactions, with Compound **4A** showing the strongest affinity. Although FQs are primarily known for their antibacterial properties, targeting bacterial DNA gyrase and topoisomerase IV [59], their interactions with other proteins, such as catalase, warrant further exploration. The presence of multiple hydrogen bonds and hydrophobic interactions points to a stable binding conformation, potentially contributing to their observed biological effects.

The structural characteristics of the FQs, including π-alkyl interactions with the heme group, suggest a mechanism for competitive inhibition. The binding of the heme group is crucial for the activation of catalase, as it plays a fundamental role in the enzyme’s catalytic activity. The heme group, which is deeply embedded in the core structure of catalase, features a pentacoordinated iron atom that is essential for its function [24,60]. This iron is coordinated by a proximal tyrosine residue, and its proper positioning is critical for the enzyme’s ability to react with H_2_O_2_ and decompose it into water and oxygen. The heme group is surrounded by a hydrophobic pocket formed by the β-barrel domain of the catalase subunit, which includes specific amino acid residues such as Met60, Ser216, Leu298, and Met349 that interact with the heme and help maintain its position. During the catalytic mechanism, the heme group serves as the site for the initial oxidation of H_2_O_2_, leading to the formation of a reactive intermediate known as compound I. This intermediate then reacts with a second molecule of H_2_O_2_ to generate oxygen and water, effectively regenerating the enzyme’s resting state [24,60,61]. Thus, the binding of the heme group ensures the correct positioning of the substrate and facilitates the rapid two-electron reduction in compound I, making it essential for the efficient decomposition of H_2_O_2_. Previous studies have indicated that FQs can induce oxidative stress in bacterial and mammalian cells, leading to potential cytotoxic effects. However, a study by Kowalska and co-workers (2020) demonstrated that lomefloxacin, a fluoroquinolone, significantly increased the activity and expression of CAT at higher concentrations, enhancing its ability to alleviate oxidative stress [62]. Similarly, the binding affinities of the FQs to CYP3A4 imply potential modulation of the enzyme’s activity, which is critical for drug metabolism and the oxidative-stress response. The lack of hydrogen bond interactions in several compounds suggests reliance on hydrophobic and π-cation interactions, potentially affecting their metabolic pathways and enhancing their antioxidant properties. The binding interactions of compound **4A** with CYP3A4, specifically through hydrogen bonding with Arg372 and π-π stacking with the heme group, suggest significant implications for its antioxidant activity. The hydrogen bond with Arg372 stabilises the compound’s binding, potentially inhibiting CYP3A4 and reducing the formation of pro-oxidant metabolites, thereby enhancing its overall antioxidant effects [63]. Meanwhile, the π-π stacking interaction with the heme may facilitate electron transfer processes essential for antioxidant activity, allowing the compound to scavenge free radicals or ROS more effectively [64]. For Keap-1, the binding affinities of the FQ compounds strongly suggest that these derivatives may modulate the Keap-1/Nrf2 signalling pathway, which represents one of the most critical cellular defence mechanisms against oxidative stress. Under basal physiological conditions, Keap-1 functions as a substrate adaptor within a Cullin3-based E3 ubiquitin ligase complex, continuously targeting Nrf2 for proteasomal degradation and thereby maintaining low cytoplasmic Nrf2 levels. This suppression is achieved through the high-affinity binding of two Nrf2 motifs, the ETGE and DLG motifs, to the Kelch propeller domain of Keap-1 [28]. Upon exposure to electrophilic compounds or oxidative signals, key cysteine sensor residues on Keap-1, particularly Cys151, Cys273, and Cys288, undergo modification, inducing conformational changes that disrupt the Keap-1/Nrf2 protein–protein interaction. This allows Nrf2 to escape ubiquitination, accumulate in the cytoplasm, translocate to the nucleus, and bind ARE in the promoter regions of target genes, driving the transcriptional upregulation of a broad antioxidant programme [65,66]. The docking results of the present study revealed that compounds 4A, 4B, and 4C form multiple hydrogen bonds with residues Arg380, Asn414, Tyr525, and Ser555, which line the binding groove of the Keap-1 Kelch domain; the same groove that accommodates the ETGE motif of Nrf2 [28]. This suggests that the FQ derivatives may act as competitive disruptors of the Keap-1/Nrf2 protein–protein interaction, mimicking the binding pose of Nrf2 itself. Importantly, this mechanism provides a compelling and explicit explanation for the apparent discrepancy between the weak docking affinities toward GPx-1 and SOD and their robust in vivo upregulation. Since GPx and SOD are well-established transcriptional targets of Nrf2/ARE-driven gene expression [56,57,58], their in vivo restoration does not require direct ligand binding to these enzymes. Rather, FQ-mediated competitive disruption of the Keap-1/Nrf2 interaction at the Kelch domain is sufficient to initiate a downstream transcriptional cascade that collectively restores the entire antioxidant enzyme network, including GPx, SOD, CAT, and GSH biosynthesis, explaining the broad-spectrum antioxidant activity observed across both experimental models. Furthermore, the lipophilic character of these novel FQ derivatives is reflected in their LogP values, which ranged from 3.43 for compound **4D** to 4.66 for compound **4E**. Notably, compounds bearing the longer n-hexyl chain at the R position (**4A** and **4E**) exhibited higher LogP values compared to their n-butyl counterparts (**4B**, **4C**, and **4D**), confirming that the n-hexyl substitution contributes more substantially to lipophilicity. All values exceed LogP 3.0, consistent with Lipinski’s criteria for favourable membrane permeability [67], and are expected to facilitate intracellular access to Keap-1 and other molecular targets, thereby supporting the robust in vivo antioxidant and hepatoprotective efficacy observed in both models. Interestingly, despite compound **4E** exhibiting the highest LogP value (4.66), compound **4A** demonstrated superior overall antioxidant activity and therapeutic efficacy against hepatotoxicity across both models. This suggests that while lipophilicity facilitates membrane permeability and target accessibility, the nature of the halogen substituent and the resulting molecular interactions, as evidenced by the docking results, are equally determinant of biological activity. The fluorine substituent in compound **4A**, known for its ability to enhance metabolic stability and modulate electronic properties [68], may contribute to its superior binding affinity and biological efficacy relative to the chloro- and bromo-analogues.

The negative binding energies and multiple hydrogen bond interactions observed particularly for compounds **4A**, **4B**, and **4C** indicate stable binding conformations at Keap-1 that could sustain inhibition of the Keap-1/Nrf2 interaction and prolong Nrf2 activation. These findings align with the growing body of literature demonstrating that small-molecule inhibitors of Keap-1 represent a promising therapeutic strategy for managing oxidative-stress-related conditions [28,65,66].

Overall, this study provided insightful findings on the therapeutic potential of FQ derivatives against hepatic injury and their promising antioxidant activity. However, the relatively small sample size (n = 4), while sufficient to determine significant differences between groups, may limit the generalisability of the findings. Future studies with larger sample sizes are needed to further validate these results. Moreover, this study focused on a set of key antioxidant parameters including GSH, CAT, SOD and GPx. While these parameters play crucial roles in the response against oxidative stress, in vivo assessment of additional parameters such as inflammatory mediators can provide valuable insights into the pharmacological activity of these derivatives.

## 5. Conclusions

In conclusion, our findings demonstrated the antioxidant activity of novel lipophilic FQs, confirmed in two oxidative-stress models. FQ derivatives exhibited ameliorative effects, alleviating the acute liver injury induced by APAP and CCl_4_ via reduction in serum ALT and AST levels. In addition, the tested FQ compounds reversed the oxidative damage induced by APAP and CCl_4_, elevating the activities of CAT, GPx, and SOD and the levels of GSH and TAS. These compounds showed similar or, in some cases, superior efficacy to the potent antioxidant, ascorbic acid. Molecular docking analyses further supported these findings, revealing strong binding affinities of the FQs, particularly compound 4A, toward catalase, CYP3A4, and Keap-1, suggesting possible enhancement of antioxidant enzyme activity, modulation of metabolic pathways, and activation of the Keap-1/Nrf2 defence mechanism. These results indicate the promising therapeutic potential of the FQ derivatives as antioxidant agents.

## Figures and Tables

**Table 1:** Targeted compounds **4** (**A**–**E**).

Compound 4	7- Substituted Aniline Derivative	R	X	Code	LogP
**4A**	4- Fluoro aniline	n-Hx	F	R 4-FACHxA	4.26
**4B**	4- Bromo aniline	n-Bu	Br	R 4-BrACBA	4.10
**4C**	4- Chloro aniline	n-Bu	Cl	R 4-ClACBA	3.83
**4D**	4- Fluoro aniline	n-Bu	F	R 4-FACBA	3.43
**4E**	4- Chloro aniline	n-Hx	Cl	R-4-ClACHxA	4.66

LogP values were calculated using ChemDraw 16.0 (PerkinElmer, Shelton, CT 06484, USA) and reflect the lipophilic character of each derivative introduced through n-hexyl and n-butyl substitutions at the R position and the type of halogen at the X position.

**Table 2:** Molecular docking targets, their PDB IDs, binding site coordinates and dimensions.

Protein	PDB ID	Binding Site Coordinates			Grid Box Dimensions (Å)		
		*x*	*y*	*z*	*x*	*y*	*Z*
CAT	1DGF	24.215	59.199	54.658	20	20	20
CYP3A4	4D6Z	19.372	27.439	−10.940	20	20	20
GPx-1	1GP1	25.078	56.439	54.911	20	20	20
IL-6	1ALU	−2.314	−13.795	1.092	20	20	20
Keap-1	2FLU	1.979	7.582	1.387	20	20	20
SOD	2C9V	17.547	−20.191	16.685	20	20	20

CAT, catalase; CYP3A4, cytochrome P450 isozyme 3A4; GPx−1, glutathione peroxidase−1; IL−6, interlukin−6; Keap−1, Kelch-like ECH-associated protein 1; SOD, superoxide dismutase.

## Data Availability

The original contributions presented in this study are included in the article/supplementary material. Further inquiries can be directed to the corresponding author.

## Supplementary Materials

The following supporting information can be downloaded at https://www.mdpi.com/article/10.3390/biom16040567/s1: Table S1. Molecular docking analyses of the four fluoroquinolone analogues (4A-4E) and ascorbic acid (control) against catalase, CYP3A4, glutathione peroxidase-1, interlukin-6, Kelch-like ECH-associated protein 1, and superoxide dismutase. Figure S1. 2D illustrations of the five fluoroquinolone analogues (4A-4E) and ascorbic acid at the binding site of glutathione peroxidase-1 (GPx-1) enzyme. Figure S2. 2D illustrations of the five fluoroquinolone analogues (4A-4E) and ascorbic acid at the binding site of interlukin-6 (IL-6). Figure S3. 2D illustrations of the five fluoroquinolone analogues (4A-4E) and ascorbic acid at the binding site of superoxide dismutase (SOD) enzyme. Figure S4. 2D illustrations of the five fluoroquinolone analogues (4A-4E) and ascorbic acid at the binding site of human erythrocyte catalase enzyme. Figure S5. 2D illustrations of the five fluoroquinolone analogues (4A-4E) and ascorbic acid at the binding site of cytochrome P450 3A4 (CYP3A4) isozyme. Figure S6. 2D illustrations of the five fluoroquinolone analogues (4A-4E) and ascorbic acid at the binding site of Kelch-like ECH-associated protein 1 (Keap-1).

## Abbreviations

The following abbreviations are used in this manuscript:

ALT	Alanine aminotransferase
APAP	Acetaminophen
ARE	Antioxidant response elements
AST	Aspartate aminotransferase
CAPSO	3-(Cyclohexylamino)-2-hydroxy-1-propanesulfonic acid
CAT	Catalase
CCl_4_	Carbon tetrachloride
CYP450-2E1	Cytochrome P450 enzyme 2E1
CYP3A4	Cytochrome P450 3A4
DMSO	Dimethyl sulfoxide
DPPH	Diphenyl-2-61 picryl-hydrazyl
FQs	Fluoroquinolones
GSH	Reduced glutathione
GPx	Glutathione peroxidase
H_2_O_2_	Hydrogen peroxide
IL-6	Interleukin-6
Keap-1	Kelch-like ECH-associated protein 1
LDH	Lactate dehydrogenase
LEB	Lowest 184 energy of binding
MM2	Molecular Mechanics 2
NADH	Nicotinamide adenine dinucleotide
NAPQI	N-acetyl-p-benzoquinone imine
NCG	Negative control group
O2•^−^	Superoxide anion
ROS	Reactive oxygen species
SOD	Superoxide dismutase
TAS	Total antioxidant status
vdW	Van der Waals
