# Supplementary material for: Antioxidant Properties of Novel Lipophilic Fluoroquinolone Compounds Against Oxidative Stress Induced by Acetaminophen and Carbon Tetrachloride in Male Wistar Rats
Source: Biomolecules. 2026 Apr 10;16(4):567. doi: 10.3390/biom16040567 (PMC13114021; doi:10.3390/biom16040567)
Supplement: Supplementary file 1 [file biomolecules-16-00567-s001.zip › biomolecules-4238325-supplementary.pdf]

Antioxidant Properties of Novel Lipophilic Fluoroquinolone Compounds Against Oxidative Stress Induced by Acetaminophen and Carbon Tetrachloride in Male Wistar Rats

Table S1. Molecular docking analyses of the four fluoroquinolone analogues (4A-4E) and ascorbic acid (control) against catalase, CYP3A4, glutathione peroxidase-1, interlukin-6, Kelch-like ECH-associated protein 1, and superoxide dismutase.

Figure S1. 2D illustrations of the five fluoroquinolone analogues (4A-4E) and ascorbic acid at the binding site of glutathione peroxidase-1 (GPx-1) enzyme.

Figure S2. 2D illustrations of the five fluoroquinolone analogues (4A-4E) and ascorbic acid at the binding site of interlukin-6 (IL-6).

Figure S3. 2D illustrations of the five fluoroquinolone analogues (4A-4E) and ascorbic acid at the binding site of superoxide dismutase (SOD) enzyme.

Figure S4. 2D illustrations of the five fluoroquinolone analogues (4A-4E) and ascorbic acid at the binding site of human erythrocyte catalase enzyme.

Figure S5. 2D illustrations of the five fluoroquinolone analogues (4A-4E) and ascorbic acid at the binding site of cytochrome P450 3A4 (CYP3A4) isozyme**.**

Figure S6. 2D illustrations of the five fluoroquinolone analogues (4A-4E) and ascorbic acid at the binding site of Kelch-like ECH-associated protein 1 (Keap-1).

Table S1. Molecular docking analyses of the four fluoroquinolone analogues (4A-4E) and ascorbic acid (control) against catalase, CYP3A4, glutathione peroxidase-1, interlukin-6, Kelch-like ECH-associated protein 1, and superoxide dismutase.

**Target**	**Compound**	**LEB (kcal/mol)**	**No. of H-Bond Interactions**	**H-Bond Interacting Residues**	**H-Bond Length (Å)[a]**	**Interactions with Heme**	**Hydrophobic Interacting Residues**
Catalase (CAT)	4A	-10.340	2	Thr361, Asp360	3.05, 2.78	π-alkyl	Val73, Phe356, Pro162, Phe161, Val74, Ile165, His364, Ala357, Pro158, Gly353
	4B	-7.702	2	Thr361, Asp360	2.92, 2.43	π-alkyl	Val73, Phe356, Asp360, Pro162, Ala357, Phe161, Ile165, His364, Gly353, Pro158
	4C	-6.397	2	Thr361, Asp360	2.92, 2.20	π-alkyl	Val73, Phe356, Asp360, Pro162, Phe161, Val74, Ile165, His364, Ala357, Pro158, Gly353
	4D	-7.595	1	Thr361	2.97	π-alkyl	Val73, Asp360, Phe356, Pro162, Phe161, Val74, Ile165, His364, Ala357, Pro158, Gly353
	4E	-7.650	2	Thr361, Asp360	3.02, 2.22	vdW[b]	Val73, Phe356, Asp360, Pro162, Phe161, Ile165, Val74, His364, Ala357, Pro158, Gly353
	Ascorbic acid	-3.830	2	Thr361, Asp360	2.84, 2.03	vdW	Val73, Ala357, Phe161, Pro162, Phe356
CYP3A4	4A	-9.572	1	Arg372	6.91	π-π stacking	Arg372, Glu374, Ala370, Met371, Phe215, Arg105, Leu373, Phe108, Phe213, Phe57, Ala305, Ser119, Ile301
	4B	-8.782	0	N/A	N/A	π-cation	Phe215, Ala370, Met371, Arg105, Phe108, Phe213, Phe57, Arg372, Leu373, Ser119, Ala305, Ile301, Phe304
	4C	-8.704	0	N/A	N/A	π-cation	Phe215, Ala370, Met371, Arg105, Phe108, Phe213, Phe57, Arg372, Glu374, Ser119, Ala305, Ile301

	4D	-8.546	0	N/A	N/A	π-cation	Phe215, Met371, Ala370, Gly481, Leu482, Thr309, Phe108, Phe57, Arg372, Arg105, Ser119, Ala305, Ile301
	4E	-9.286	0	N/A	N/A	π-cation	Phe215, Ala370, Met371, Arg105, Phe108, Phe213, Phe304, Phe57, Arg372, Leu373, Ser119, Ala305, Ile301
	Ascorbic acid	-4.531	1	Phe304	3.65	π-donor H-bond	Ala305, Thr309, Arg212, Ala370, Phe213
Glutathione Peroxidase-1 (GPx-1)	4A	-4.656	1	Arg177	5.88	N/A	Arg178, Arg50, Thr47, Se745, Trp158, Leu180, Asp134, Phe179
	4B	-4.191	2	Arg178, Arg177	4.08, 5.85	N/A	Arg50, Arg178, Thr47, Gly46, Leu180, Phe179, Asp134
	4C	-4.328	2	Arg178, Arg177	4.34, 5.94	N/A	Arg178, Arg50, Thr47, Gly46, Leu180, Phe179, Asp134
	4D	-4.086	1	Arg177	5.91	N/A	Arg178, Arg50, Thr47, Arg177, Leu180, Phe179, Asp134
	4E	-4.650	3	Arg178, Arg178, Arg177	3.87, 5.29, 5.99	N/A	Arg178, Arg50, Thr47, Arg177, Se745, Trp158, Leu180, Phe179, Asp134
	Ascorbic acid	-4.947	4	Leu139, Ser157, Arg177, Arg178	5.70, 4.43, 5.09, 2.51	N/A	Ala138, Pro132, Asp135, Ser176, Met140, Thr141, Trp158
Interleukin-6 (IL-6)	4A	-5.681	3	Arg179, Arg182, Arg182	2.80, 6.34, 5.08	N/A	Arg179, Arg182, Gln175, Leu178, Leu33, Arg30, Asp34
	4B	-5.805	4	Arg182, Arg182, Arg179, Arg179	5.78, 4.78, 3.13, 2.92	N/A	Arg179, Gln175, Leu178, Arg30, Leu33, Asp34
	4C	-5.853	3	Arg179, Arg182, Arg182	2.88, 5.00, 5.59	N/A	Arg179, Gln175, Leu178, Leu33, Arg30, Asp34
	4D	-5.562	3	Arg179, Arg179, Arg183	2.85, 3.04, 5.11	N/A	Arg179, Gln175, Leu178, Leu33, Arg30, Asp34
	4E	-5.656	4	Arg182, Arg182, Arg179, Arg179	5.60, 5.03, 3.23, 2.83	N/A	Arg179, Arg182, Gln175, Leu178, Leu33, Arg30, Asp34

	Ascorbic acid	-4.587	5	Arg182, Arg179, Arg179, Asp26, Gln175	4.77, 2.39, 2.87, 4.74, 4.11	N/A	Leu178, Arg30, Ser176
Kelch-like ECH-associated protein 1 (Keap-1)	4A	-7.968	4	Arg380, Arg380, Asn414, Tyr525	3.66, 5.38, 3.93, 5.82	N/A	Ala556, Arg415, Arg483, Tyr572, Ser602, Phe577, Tyr334, Asn382, Gly364, Ser363, Gly603, Ser555, Gln530, Tyr525
	4B	-8.086	3	Arg380, Asn414, Ser555	5.44, 4.51, 4.22	N/A	Ala556, Arg415, Arg483, Tyr572, Ser602, Phe577, Gly364, Ser363, Gly603, Gln530, Tyr525
	4C	-7.481	3	Arg380, Arg380, Ser555	2.91, 5.41, 4.28	N/A	Ala556, Arg415, Asn414, Arg483, Tyr572, Ser602, Phe577, Gly364, Ser363, Gly603, Gln530, Tyr525
	4D	-7.891	3	Arg380, Arg380, Ser555	2.80, 5.36, 4.22	N/A	Ala556, Arg415, Asn414, Arg483, Tyr572, Ser602, Phe577, Gly364, Ser363, Gly603, Gln530, Tyr525
	4E	-7.810	1	Arg380	5.37	N/A	Ala556, Arg415, Asn414, Arg483, Tyr572, Ser602, Phe577, Tyr334, Asn382, Gly364, Ser363, Gly603, Ser555, Gln530, Tyr525
	Ascorbic acid	-5.574	4	Ser363, Ser363, Arg380, Arg415	3.42, 4.73, 3.31, 3.46	N/A	Tyr334, Asn382, Arg415, Gly364, Asn414, Ala556, Gly603, Ser602, Ser338
Superoxide Dismutase (SOD)	4A	-5.897	4	Lys136, Lys136, Lys136, Arg69	5.15, 4.12, 3.86, 7.11	N/A	Pro62, His80, Lys136, Asn65, Thr137, His63, Lys70, Thr135, Ser68, Gly61, Arg143, Thr58
	4B	-6.154	2	Lys136, Lys136	3.98, 5.05	N/A	Pro62, His80, Lys136, Asn65, Thr137, His63, Arg69, Lys70, His71, Thr135, Arg143, Gly61, Thr58
	4C	-6.137	2	Lys136, Lys136	3.99, 5.22	N/A	Pro62, His80, Lys136, Asn65, Thr137, His63, Arg69, Lys70, His71, Thr135, Arg143, Gly61, Thr58
	4D	-5.909	4	Lys136, Lys136, Lys136, Lys136	4.35, 4.01, 5.76, 5.21	N/A	Pro62, His80, Lys136, Asn65, Thr137, His63, Arg69, Lys70, His71, Thr135, Arg143, Gly61, Thr58

4E	-6.588	2	Lys136, Lys136	4.01, 5.06	N/A	Pro62, His80, Lys136, Asn65, Thr137, His63, Arg69, Lys70, His71, Thr135, Arg143, Gly61, Thr58
Ascorbic acid	-4.641	4	His63, Asn65, His80, His80	2.52, 3.38, 4.21, 5.65	N/A	Lys136, Thr137, Pro62, Arg69, Ser68, Lys70, His71, Thr135

a: H-bond interactions lengths are presented in order of the H-bond interacting residues.

b: vdW, van der Waals binding interactions.

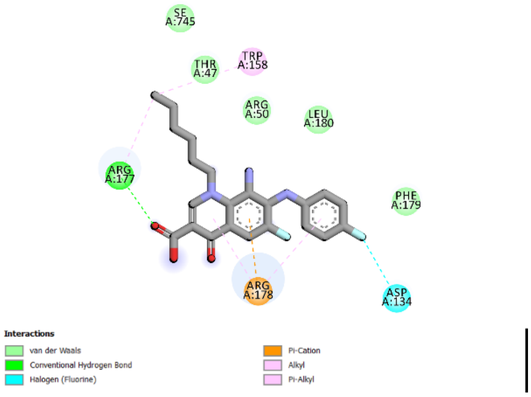

4A

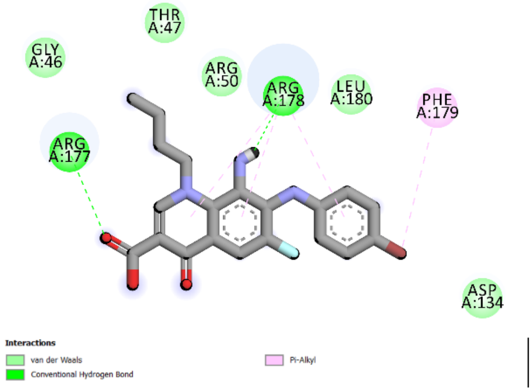

4B

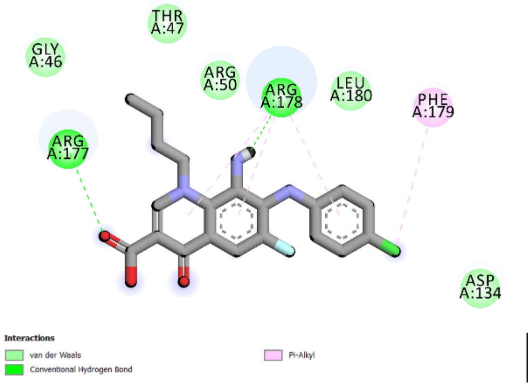

4C

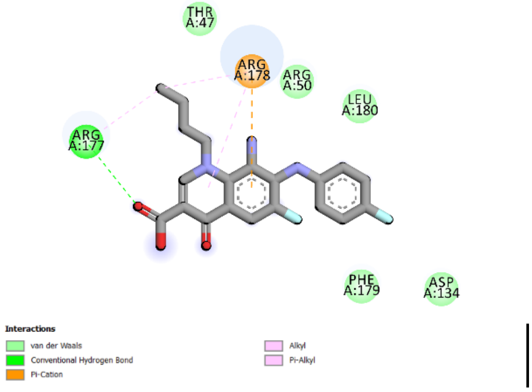

4D

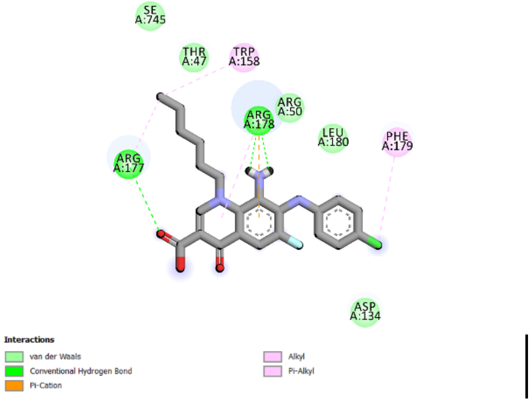

4E

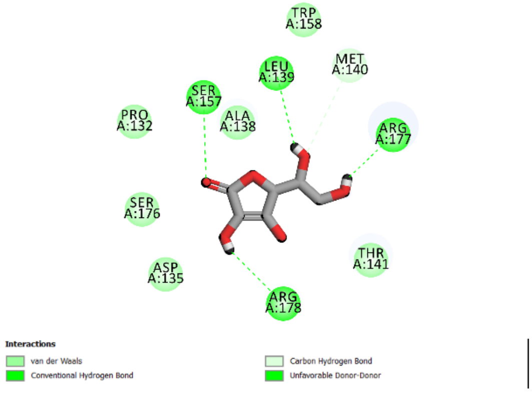

Ascorbic Acid

Figure S1. 2D illustrations of the five fluoroquinolone analogues (4A-4E) and ascorbic acid at the binding site of glutathione peroxidase-1 (GPx-1) enzyme.

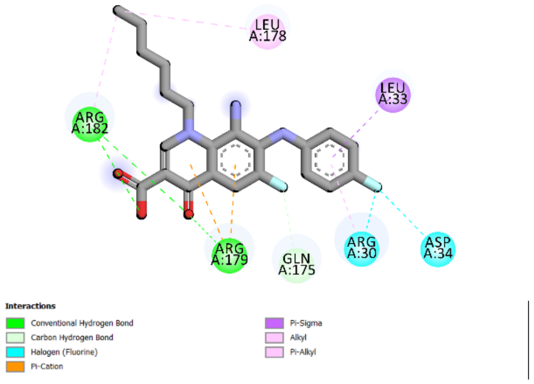

4A

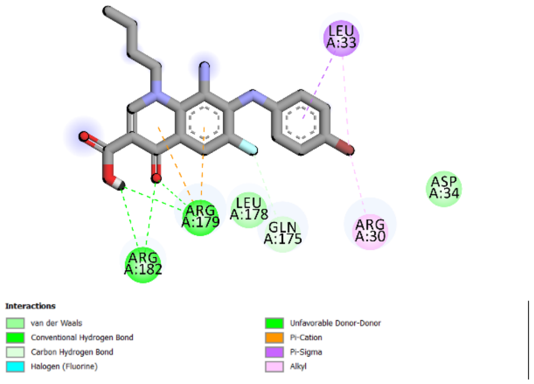

4B

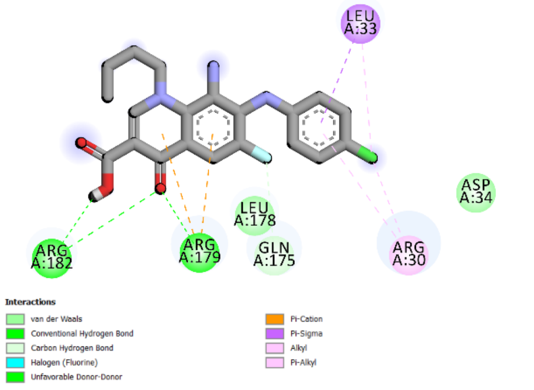

4C

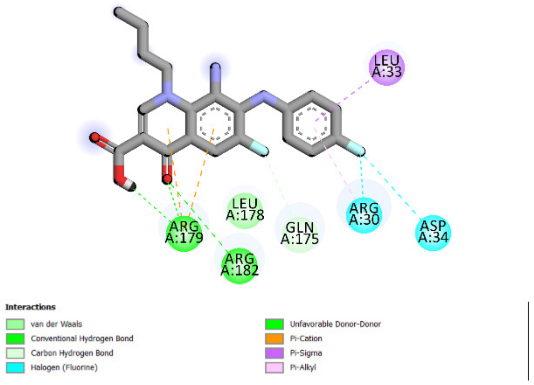

4D

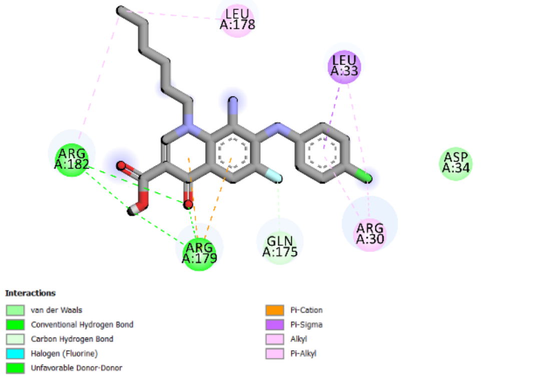

4E

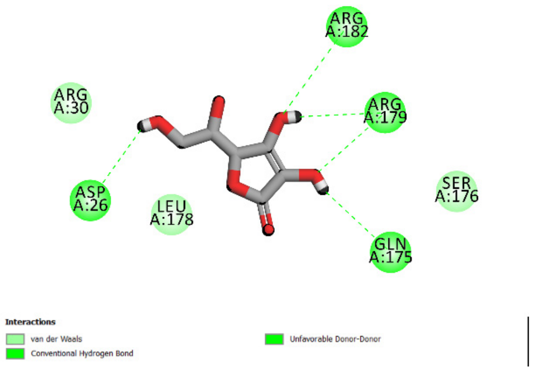

Ascorbic Acid

Figure S2. 2D illustrations of the five fluoroquinolone analogues (4A-4E) and ascorbic acid at the binding site of interlukin-6 (IL-6).

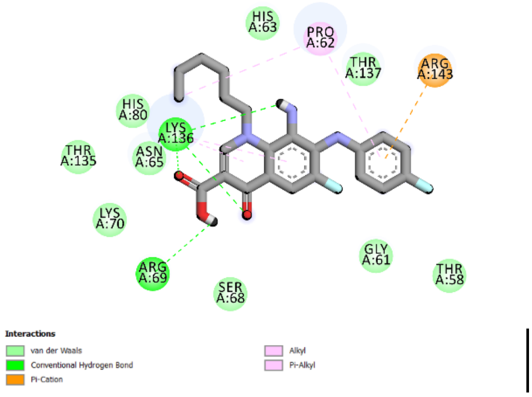

4A

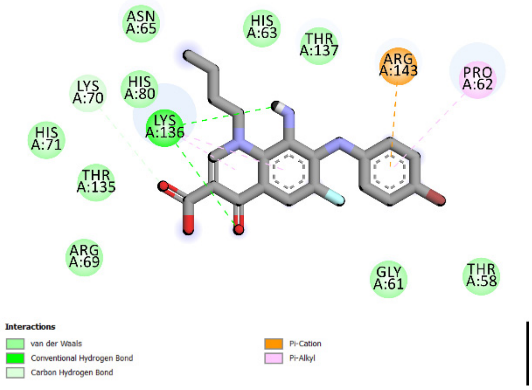

4B

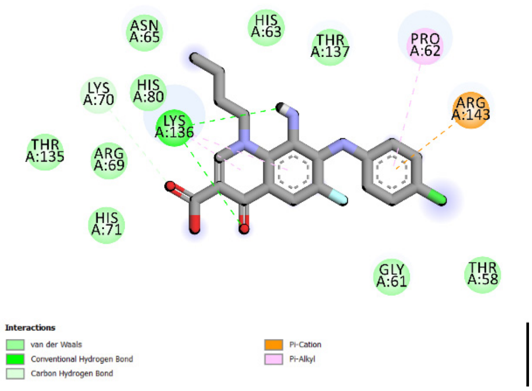

4C

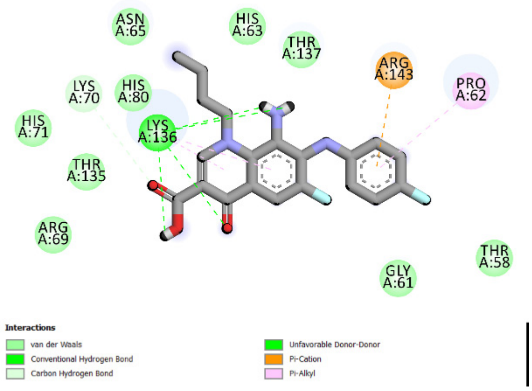

4D

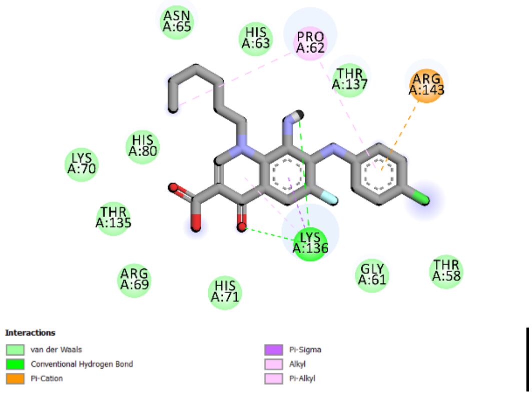

4E

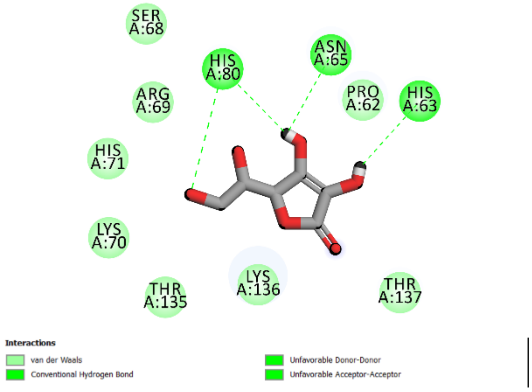

Ascorbic Acid

Figure S3. 2D illustrations of the five fluoroquinolone analogues (4A-4E) and ascorbic acid at the binding site of superoxide dismutase (SOD) enzyme.

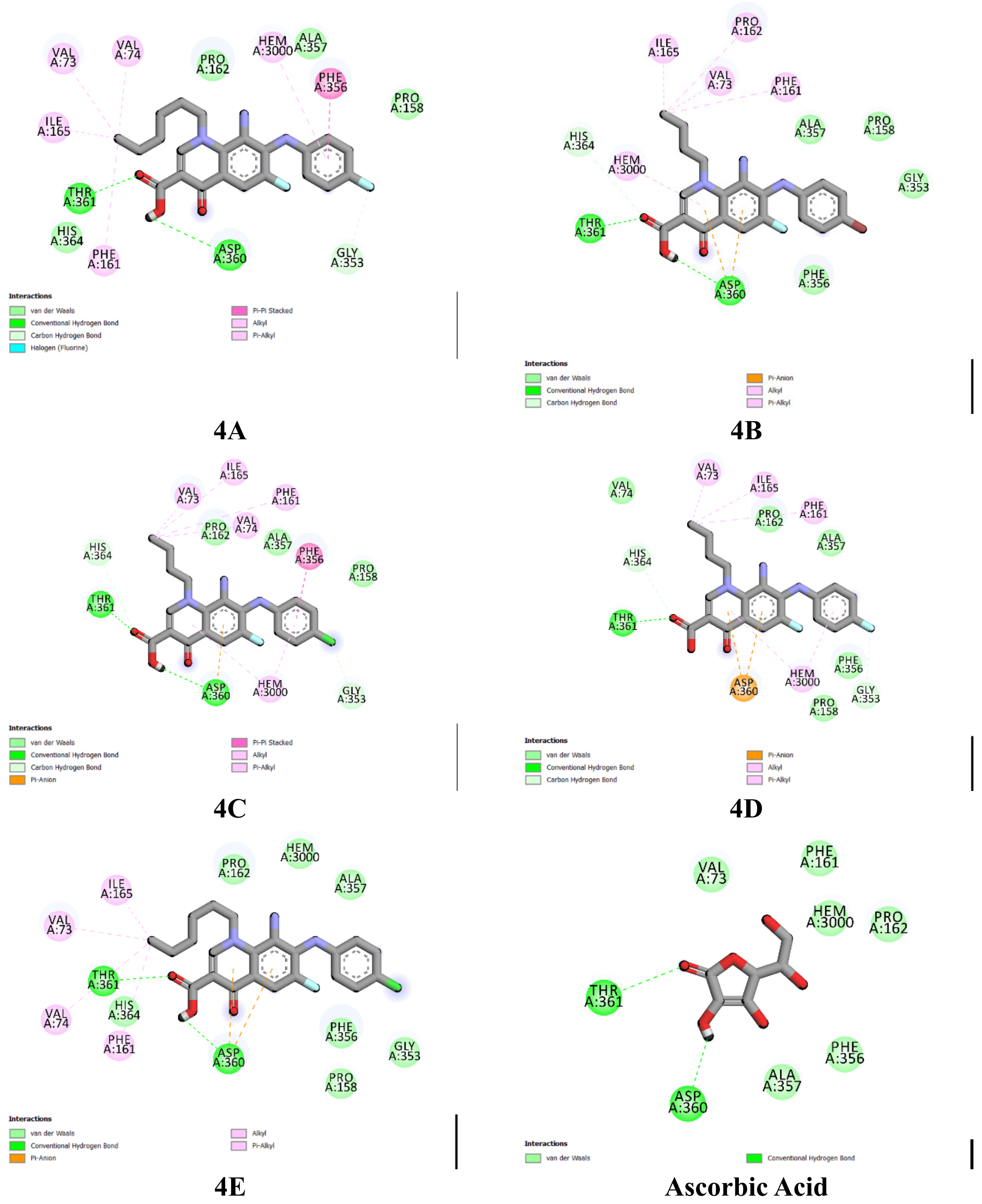

Figure S4. 2D illustrations of the five fluoroquinolone analogues (4A-4E) and ascorbic acid at the binding site of human erythrocyte catalase enzyme.

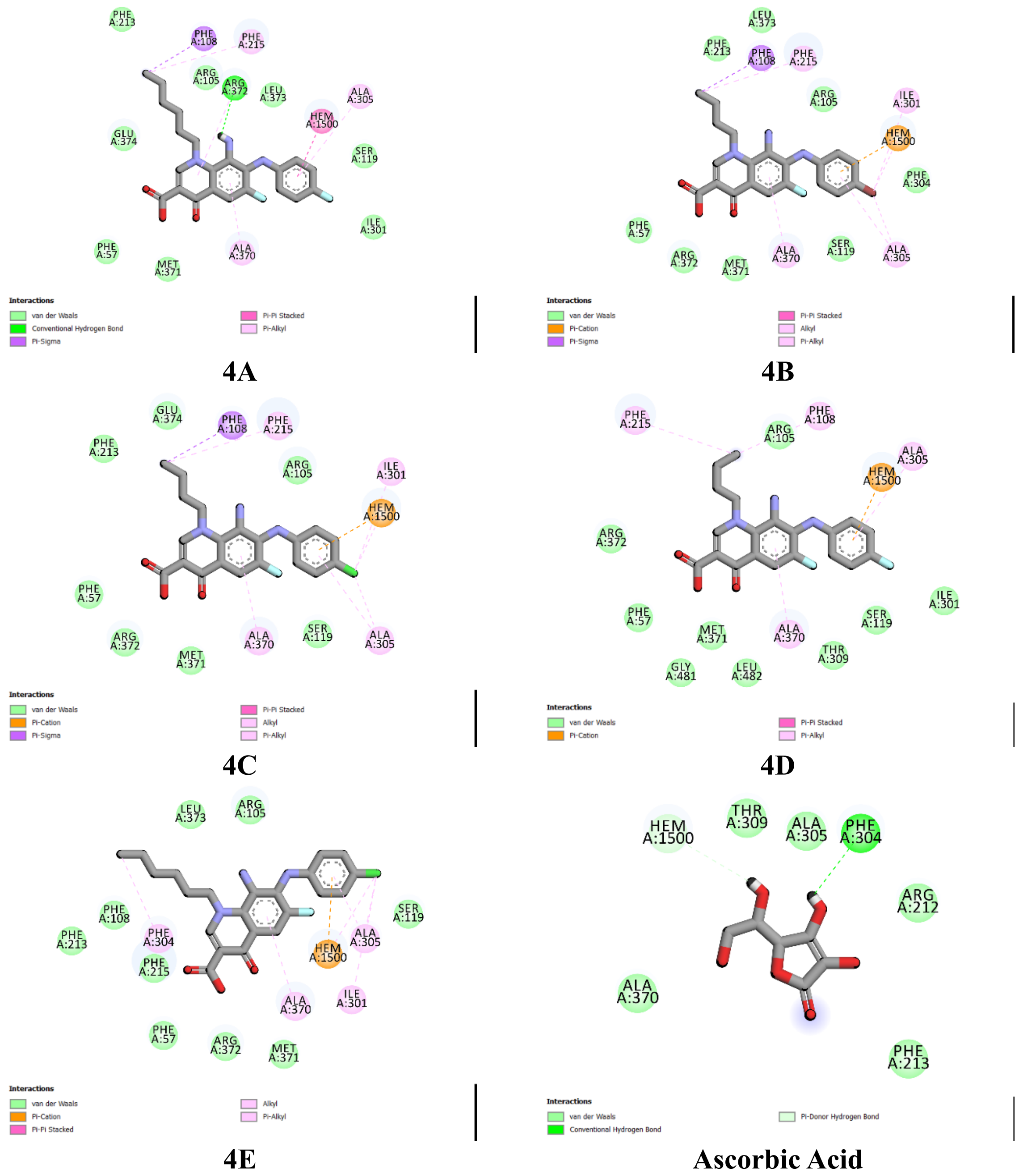

Figure S5. 2D illustrations of the five fluoroquinolone analogues (4A-4E) and ascorbic acid at the binding site of cytochrome P450 3A4 (CYP3A4) isozyme.

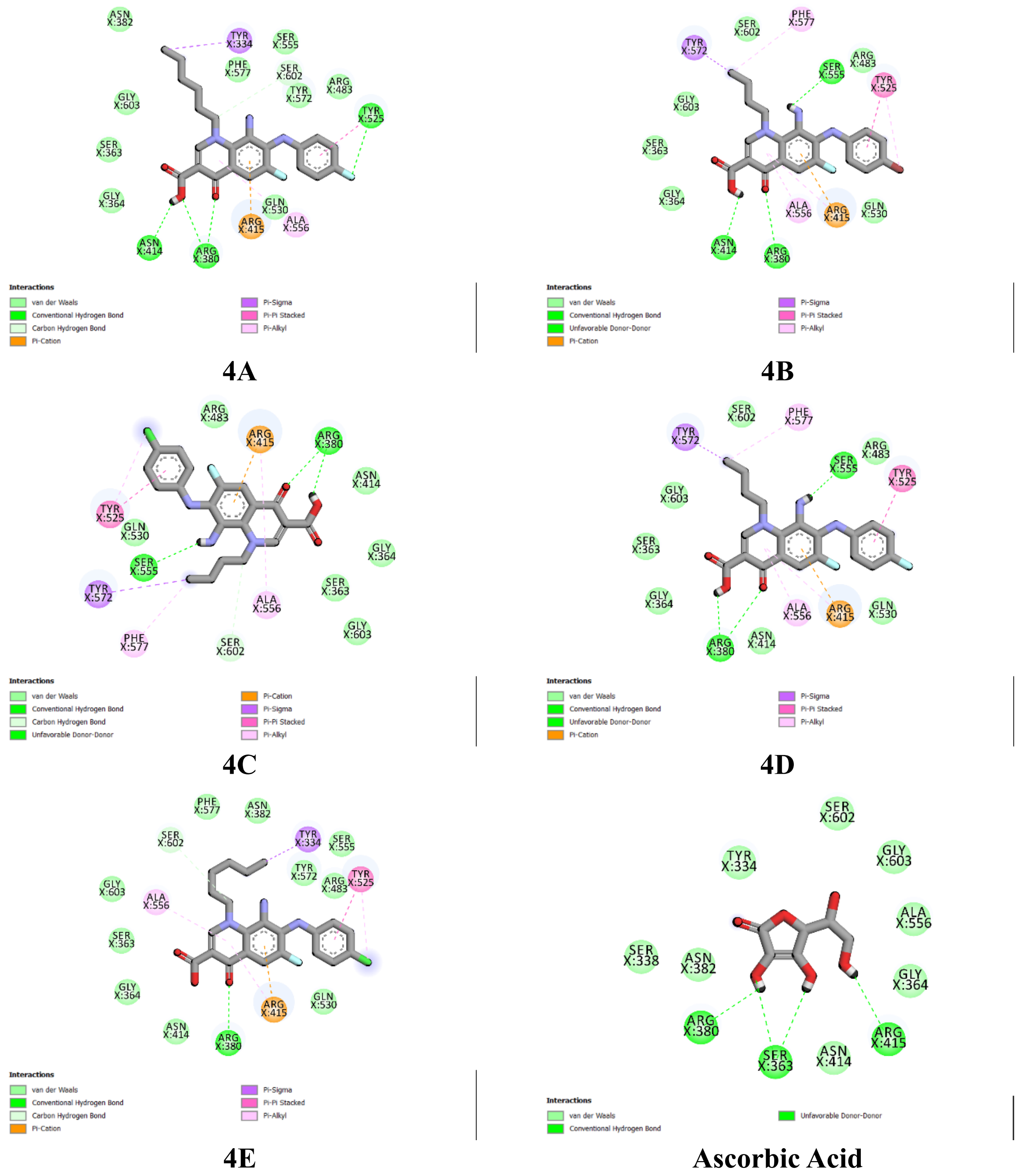

Figure S6. 2D illustrations of the five fluoroquinolone analogues (4A-4E) and ascorbic acid at the binding site of Kelch-like ECH-associated protein 1 (Keap-1).
